# Early effect of different bifurcation techniques on left ventricular mechanics in elective percutaneous coronary intervention

**DOI:** 10.1186/s43044-024-00502-5

**Published:** 2024-07-02

**Authors:** Amr Nasser Elsheikh, Ayman Elsaeid, Samia Sharafeldin, Sahar Elshedoudy, Ehab ElGendy

**Affiliations:** Cardiology Department, Tanta University, 55-ElGish Street, Tanta, Gharbia Egypt

**Keywords:** Bifurcation techniques, LV mechanics, Percutaneous coronary intervention

## Abstract

**Background:**

Bifurcation lesions are prevalent amongst patients with symptomatic coronary artery disease subjected to percutaneous coronary intervention (PCI). Recent consensus commends a conservative (provisional) approach when managing the side branch. Here, the aim was to explore the immediate impact of different bifurcation techniques (one stent and two stent strategies) on left ventricular LV) myocardial functions using speckle tracking echocardiography in patients subjected to elective PCI. Sixty two consecutive patients diagnosed with coronary bifurcation lesion (CBL) were enrolled. Patients were categorized into: one-stent strategy (Provisional group, *n* = 44) and a two-stent strategy (TAP, DK crush, or Culotte technique, *n* = 18), based on the coronary bifurcation site, angle, side branch diameter and Medina classification. LVEF%, regional and global longitudinal strain (GLS), and E/E' were measured before and within 24 h post PCI.

**Results:**

In both provisional and 2- stent technique, the mitral inflow velocities and mitral annular velocities showed improvement with significant reduction in E/e' (*P* < 0.03 and *P* < 0.001) respectively while LVEF% did not change. There were no significant changes in any other echo parameters post PCI. In provisional group, there were significant improvements in LAD (*P* < 0.001), RCA (*P* < 0.01) territories and GLS (*P* < 0.01). Δ LAD was expressively higher (34.5%) compared with **Δ** LCX (9.6%) and ΔRCA (25.4%), *P* < 0.001, *P* < 0.01 respectively. In the 2-stent technique group, there were significant improvements in peak longitudinal strain of LAD territory (*P* < 0.01), RCA territory (*P* < 0.01) and GLS (*P* < 0.01) respectively. **Δ** LAD territory was significantly higher in provisional group in comparison with the 2- stent technique group. ***Δ*** GLS was correlated inversely to Gensini score in provisional group and to the number of vessel diseased in 2-stent technique group.

**Conclusion:**

PCI of the bifurcation lesion positively impact myocardial function. Both bifurcation techniques improve LV mechanical properties using 2D strain imaging while LV EF% remains unchanged.

## Background

Coronary bifurcation lesions (CBL) are not uncommon in patients presenting with symptomatic coronary artery disease (CAD). Approximately 15% to 20% of lesions treated by percutaneous coronary intervention (PCI) are coronary bifurcation lesions (CBL) [1]. Bifurcation lesions remain difficult for interventional cardiologists to treat, even with significant developments in stent technology. Treatment is linked to higher rates of long-term restenosis, periprocedural myocardial infarction (MI), stent thrombosis, and additional cost [2, 3]. The current standard of therapy is the conservative (provisional) strategy, which treats the main branch (MB) first and the side branch (SB) only if necessary, despite the fact that other tactics and procedures have been offered [4–10]. Nevertheless, conclusive findings in previous research have been hampered by recruitment bias, limited sample numbers, varied PCI procedures, and the absence of stringent outcomes [2, 3, 11–14]

A bare-metal stent called a "dedicated bifurcation stent" that is intended to treat and secure the bifurcation SB has recently shown promising results in a number of short, nonrandomized trials [7–11] The TRYTON trial was a prospective, multicenter, single-blind, randomized, controlled study that assessed the efficacy and safety of the Tryton side branch stenting with DES in treating de Novo CBL in the Main Branch & Side Branch in Native Coronaries compared to SB balloon angioplasty [7–11] While Tryton may be used in complicated bifurcation anatomies with severe illness in big side branches, the provisional technique should still be regarded the default approach in most bifurcation instances.

The double kissing (DK) crush planned 2-stent technique produced lower rates of target lesion revascularization (TLR), stent thrombosis (ST), and composite major adverse cardiac events compared with culotte stenting in distal LM bifurcation lesions [3, 4], and lower rates of TLR compared with provisional stenting (PS) in non-LM coronary bifurcation lesions [14]. However, no research has directly contrasted the impact of various methods for treating bifurcation lesions with respect to heart function.

We attempt to comprehensively evaluate the impact of the different bifurcation lesions techniques on left ventricular (LV) mechanical function using two- dimensional strain imaging in patients undergoing PCI. In the current study we examined the immediate consequence of different bifurcation techniques and comparative outcome of LV systolic and diastolic mechanics using 2D- strain echocardiography.

## Methods

### Study population

This was a single center, observational cross- sectional study. Between February 2021 and June 2023, 101 patients with coronary bifurcation lesions were enrolled from cardiology department Tanta university and were subjected to elective PCI.

Patients with one of the following criteria were included in the study: chronic coronary artery disease [including chronic coronary syndrome (CCS) or history of acute coronary syndrome (ACS) > one month] undergoing PCI to de novo bifurcation lesions, with > 50% diameter stenosis (DS) of the main branch (MB) and/or side branch (SB) with different techniques according to operator preference.

Coronary bifurcation lesions included: left main (LMCA), Left anterior descending- Diagonal (LAD-D), Left circumflex- Obtuse marginal (LCX-OM) and Posterior descending- posterolateral (PDA-PL) coronary arteries.

Patients with one or more of the following were excluded from the study: ACS, acute heart failure, prior coronary artery bypass graft (CABG), complicated PCI e.g. MI, perforation, no reflow, cardiac arrest, failed PCI, poor echocardiographic window. The study protocol was permitted by institutional review board and ethical committee of the faculty of medicine, Tanta University, and all procedures followed the principles of the Declaration of Helsinki. All patients provided written informed consent prior to their participation in the study.

Of the 101 patients diagnosed with CBL, 62 patients (56.4%) were involved in the study. Patients were categorized into two groups according to the PCI strategy: a one-stent strategy (Provisional group, *n* = 44) and a two-stent strategy group (TAP, DK crush or Culotte technique, *n* = 18). The PCI strategy was based on the bifurcation site, angle, SB diameter and Medina classification [15, 16].

Every patient had, in addition to transthoracic echocardiographic examination, a comprehensive clinical examination, a 12-lead ECG, and a history taking.

### Echocardiographic measurements

Using Vivid 9 (General Electric-Vingmed and Horton, Norway), conventional and two-dimensional (2D) strain images were acquired from standard parasternal and apical perspectives. The American Society of Echocardiography's guidelines were followed for taking LV measures. [17, 18] Left atrial diameter (LAD), left ventricular end-diastolic and end-systolic diameters (LVDD and LVSD in mm), interventricular septum (IVS), posterior wall (PW) thickness at end-diastole, and left ventricular mass indexed to body surface area (LVMI) were all measured using M-Mode echo with 2-D guidance [17, 18] We used Simpson's biplane approach to determine the LV ejection percentage (EF%). The pulsed-wave Doppler was used to record the mitral inflow velocities in order to quantify the following parameters: isovolumetric, E/A ratio, peak early (E) and late (A) diastolic velocities, and deceleration time of E velocity (DT) and isovolumetric relaxation time (IVRT). All measurements were derived from the average of at least three consecutive cardiac cycles.

### Tissue Doppler measurements

Mitral annular velocity was measured by pulsed wave tissue Doppler imaging (TDI) from the apical 4-chamber view and averaged from the lateral and medial annuli. The resulting velocities were obtained from 3 consecutive cardiac cycles at a sweep speed of 50 mm/s. The peak early (*E*′) diastolic annular velocity was measured and the *E*/*E*′ ratio was used to estimate LV filling pressures. E/E' ratios > 8 indicated elevated LV end diastolic pressures.

Echocardiographic studies were recorded by single operator to reduce interobserver variability.

### 2D strain imaging

Speckle tracking echocardiography was performed at the time of admission (pre-intervention) and postintervention before the patient's hospital discharge.

Measurement of peak longitudinal strain parameters utilized speckle tracking to evaluate cardiac mechanics and quantify regional and global longitudinal strain (GLS) from apical views. The GLS average (GLS-Avg) was calculated from 17 myocardial segments, from the 3 longitudinal planes, (apical 4, 2 and 3 long axis views) i.e., vertically and basal, mid, and apical, i.e. horizontally of the anterior, anteroseptal, inferoseptal, inferior, inferolateral, and anterolateral walls according to the current standards [18–20].

Every image under analysis was captured at a frame rate greater than 55 frames per second utilizing the Echo Pack system version 110.0.x (GE Medical System), and data were moved for offline analysis [19] Using the perfusion territories of the three major coronary arteries as a basis, a 17-segment left ventricular model's territorial longitudinal strain (TLS) was computed by averaging the segmental peak systolic strain values inside each territory [18, 19].

For comparison with the angiographic findings: basal anterior and anteroseptal, mid-anterior and anteroseptal, apical anterior, septal, and apex were representing the LAD distribution; basal inferoseptal and inferior, mid-inferoseptal and inferior, and apical inferior were assigned to the RCA; and basal inferolateral and anterolateral, midinferolateral and anterolateral, and apical lateral were assigned to the LCX.

To calculate the magnitude of change in LV strain (Δ) difference between pre and post PCI values was divided by the baseline value.

### Coronary intervention

Commercially available Siemens Cath lab devices were utilized. All patients were given an oral loading dose of P2Y12 inhibitor, IV unfractionated heparin prior PCI. Index coronary angiography procedures were performed via the femoral or radial approach using standard protocol and multiple angles. PCI to the coronary arteries with bifurcation lesions were performed using one stent and two stents- strategies. The dual-stenting techniques were performed according to the operator's decision including the following:

- *T-stenting and small protrusion (TAP):* a second stent is advanced through the struts of the MB stent into the SB and deployed with slight (1–2 mm) protrusion into the MB, then both the MB balloon and the SB stent balloon are simultaneously inflated.
- *Culotte technique*: 2 stents are deployed at the same time, from the main vessel into each branch with strut opening to each branch by kissing balloon inflation leaving the proximal main vessel covered with two overlapped stents.
- *DK Crush technique:* which consists of stenting from the main vessel into the SB, balloon crushing from the MB, kissing balloon inflation, stenting from the main vessel into the MB and final kissing balloon inflation [21–23].

Successful PCI was defined if Thrombolysis in Myocardial Infarction (TIMI) is grade 3 flow, the residual stenosis is below 20% and no procedure complications like MI, stroke, death or need of urgent CABG. [11]

### Statistical analysis

The data were shown as mean ± SD or as numbers (%).the Fisher's exact test or the Chi-Square test were used to assess the distribution of qualitative variables. A two-tailed Students' test was used to compare the means of normally distributed data. The Wilcoxon rank-sum test or the Student's t-test were used to assess continuous variables. The Pearson correlation coefficient was utilized to determine the association between the changes in LV strain values in addition to clinical and other echocardiographic data.

*P* < 0.05 was considered statistically significant. Statistical analysis was done by IBM SPSS statistical software package for MAC, version 23.

## Results

From 101 patients who were diagnosed with CBL located in a de novo native coronary artery, 21 patients were referred for CABG (9 patients had LM SYNTAX > 22; 12 patients had 3 vessel disease and SYNTAX > 22), 1 patient complicated with MI; 1 patient complicated with perforation; 16 patients did not complete the echocardiographic study post PCI. A total of 62 consecutive patients who met the inclusion criteria with complete echocardiographic and angiographic data were included in the data analysis.

Of the 62 patients with CBL, 18 patients (29%) were treated with the two-stent strategy and 44 patients (71%) were treated with provisional technique.

### Baseline clinical and demographic data

Clinical and demographic characteristics of the studied groups are depicted in (Table 1). The cohort mean age was 57 years (ranged from 45 to 70) and 42 (67.7%) were males. Patients in the 2-stent technique group had male predominance compared to the provisional group (72% versus 66%), *P* < 0.001. There were no significant differences between both groups in age, body mass index, HR, SBP or DBP. Overall, STEMI was the most common history in both groups with higher predominance in the 2-stent techniques group (76% versus 54%). Unstable angina was more prevalent in provisional group compared with 2-stent technique group (14% versus 5%), *P* < 0.001.

**Table 1:** Demographic and clinical data

Demographic and clinical data	Provisional (*N* = 44)	2 Stent technique	p-value
		(*N* = 18)	
Age (years)	58.4 ± 6.9	56.8 ± 8.78	0.519
*Gender*			
Male	29 (66%)	13 (72%)	< 0.001
Female	15 (34%)	5 (28%)	
HR	75.9 ± 11.5	75.5 ± 10.94	0.632
DBP	74.7 ± 10.1	75.4 ± 10.33	0.361
SBP	120.18 ± 13.56	121.35 ± 13.94	0.856
Weight	88.4 ± 12.6	92.5 ± 51.3	0.199
Height	1.80 ± 2.55	2.05 ± 5.12	0.303
BMI Obesity (≥ 30 kg/m2)	31.7 ± 9.1 9 (20.4%)	33.4 ± 3.3 4 (22.2%)	0.239 0.132
*PCI technique*			
Provisional	44 (100%)		
2 stent technique			
DK crush		(27.8%)5	
Culotte		4 (22.2%)	
TAP		9 (50%)	
*History of CAD*			
STEMI	24 (54.5%)	12 (66.7%)	0.001
NSTEMI	12 (27.3%)	4 (22.2%)	0.135
Unstable Angina	6 (13.6%)	1 (5.5%)	0.01
Chronic stable angina	2 (4.5%)	1 (5.5%)	0.142
*Risk factors*			
*BMI*			
Underweight < 18.5	3(6.8%)	1 (5.5%)	0.132
Normal weight 18.5–24.9	6(13.6%)	1(5.5%)	
Overweight 25–29.9	26 (59%)	13(72.3%)	
Obesity ≥ 30	9(20.0%)	3(16.7%)	
Family history of premature CAD	10(22.7%)	4(22.2%)	0.231
*Tobacco use status*			
Never	11 (25.0%)	5(27.8%)	0.432
Ex-smoker	10(22.7%)	7(38.8%)	0.01
Current smoker	23(52.3%)	6(33.3%)	0.001
Hypertension	27(61.4%)	12(66.7%)	0.122
Diabetes mellitus	26(59%)	13(72.2%)	0.01
Dyslipidemia	19(43.1%)	7(38.9%)	0.342
Type of LV dysfunction			
E/e' (> 8)	34(77.3%)	13(72.2%)	0.542
Systolic (EF < 50%)	9(20.4%)	4(24.5%)	0.432

HR: heart rate; SBP: systolic blood pressure; DBP: diastolic blood pressure; BMI: body mass index; STEMI: ST elevation myocardial infarction; NSTEMI: non-ST elevation myocardial infarction; E/e': early diastolic mitral inflow velocity to early mitral annular diastolic velocity ratio

### Patient risk profile

Nearly more than 70% of the patients in each group were clinically overweight and obese, Table 1. In the overall cohort, overweight was prevalent in 63%, while obesity exists in 20% of patients referred for PCI. Hypertension and DM were equally prevalent in overall patients 63%, 66% respectively while dyslipidemia was prevalent in 50% of studied population.

There were no significant differences between groups in prevalence of obesity, family history of CAD, dyslipidemia, or hypertension. Diabetes was present in 72% of the 2-stent technique group which was significantly higher compared to the provisional group (52%, *P* < 0.001), while current smoking was more prevalent in the provisional group compared with the 2-stent technique group (52% versus 33%, *P* < 0.001).More than 70% of patients in each group had diastolic dysfunction as estimated by E/e', and 20–25% had LV ejection fraction < 50%. There were no significant differences between groups in prevalence of systolic or diastolic dysfunction.

### Laboratory findings

The patients in the provisional group had more normal ECG, more anterior ischemia while the two-stent technique group had more frequent anterior and inferior myocardial infarction evidence on ECG compared with provisional group (*P* < 0.001). There were no relevant differences between groups in other laboratory findings.

### Medications in studied groups

Comparing patients in provisional group and 2-stent technique group, no significant differences were observed between groups regarding the prescribed medications (B blockers, ASA, P2Y12i, Nitrates, Statins, P = 0.147) at time of presentation to Cath lab.

#### Coronary angiographic findings

Diagnostic coronary angiographic data is illustrated in (Table 2). Femoral access was used in ~ 60% while radial approach was applied in ~ 40% of both studied groups.

**Table 2:** Coronary angiographic data in studied group

Arterial access site	Provisional (*N* = 44)	2 Stent technique	× 2	p-value	
		(*N* = 18)			
Arterial access site					
Femoral	26(59%)	11(61%)	1.035	0.309	
Radial	18(41%)	7(39%)			
Dominance					
Co-dominance	2(4.5%)	1(5.5%)	3.034	0.386	
LCX	2(4.5%)	0(0.0%)			
Left	1(2.2%)	1(5.5%)			
Right	39(88.6%)	16(88.8%)			

	Total (*N* = 62)	Provisional (*N* = 44)	2 Stent technique (*N* = 18	× 2	p-value
LM significant lesion	5(8.1%)	2 (4.5%)	3 (16.7%)	0.103	0.618
LAD diseased					
Normal Ectasia/atherosclerosis	2(3.2%)	2(4.5%)	0(0.0%)	3.3	0.342
	1(1.6%)	0(0%)	1(0%)		
Significant lesion	46(74.2%)	32 (72.7%)	14 (77.8%)		
LCX					
Normal	16(25.8%)	11(25%)	5(27.8%)	1.914	0.545
Ectasia/atherosclerosis	12(19.4%)	9(20.4%)	3(16.7%)		
Significant lesion	0 (0%)	21 (47.7%)	9(50%)		
RCA					
Normal	16(1%)	12(27.2%)	4(22.2%)	39.925	< 0.001
Ectasia/atherosclerosis	14(0%)	11(25%)	3(16.7%)		
Significant lesion	32 (0%)	21 (47.7%)	11(61.1%)		
No. of vessels					
1 vessel diseases	28(45.2%)	22(50%)	6 (33.3%)	20.637	< 0.001
2 vessels diseases	20(32.3%)	13(29.5%)	7 (38.9%)		
3 vessels diseases	14(22.5%)	9 (20.5%)	5 (27.7%)		
GENSINI score	17.3 ± 22.1	25.4 ± 24.2	− 3.763	< 0.001	

LAD: left anterior descending artery; LCX: left circumflex; RCA: right coronary artery

Left main disease (≥ 50% stenosis) bifurcation lesion was present in 5 (8.1%) of the overall cohort, 72% of the provisional group and 77% in 2-stent technique group, LCX disease existed in 47% and 50% of provisional and 2-stent techniques groups respectively. Significant RCA lesions were detected more frequent in 2-stent technique group, 61% compared to 45% in the provisional group.

Single vessel disease was present in 45% of study cohort while two or 3 vessel disease combined was present in 55.5% of the overall cohort with patients.

While single vessel disease was the most prevalent in the provisional group (50%) compared to 33% in the 2- stent technique group, two and three vessel disease were the least common (29% versus 39% and 20% versus 28% in the 2-Stent technique group (*P* < 0.0001) and the provisional group respectively. Accordingly, the severity of CAD in CBL cohort calculated by Gensini score was significantly higher in the provisional group compared to the 2-Stent technique group (17.3 ± 22.1 versus 25.4 ± 24.2 vs, *P* < 0.001) respectively.

Drug-eluting stents were used in all patients (100%). Most of the patients had CBL as a target lesion intervention except in 3 patients, two of them in provisional group, where PCI was directed to the more severe lesion before the bifurcation lesion intervention.

### Target vessel intervention

The target vessel intervention of CBL in provisional technique consisted of 2 LM, 30 LAD, 8 LCX and 4 RCA. In 2-stent technique the target vessel intervention included 3 LM, 12 LAD, 2 LCX and 1 RCA (Table 3).

**Table 3:** Target vessel in PCI

PCI	Provisional (*n* = 44)				2 Stents technique (*n* = 18)			
	No	(%)	Type of stents	No. of stents	No	(%)	Type of stents	No. of stents
LM	2	(4.6%)	DES	2	3	(16.7%)	DES	6
LAD	30	(68.1%)	DES	30	12	(66.7%)	DES	12
LCX	8	(9.1%)	DES	8	2	(11.1%)	DES	2
Diagonal			DES				DES	12
OM			DES				DES	2
RCA	4	(18.2%)	DES	4	1	(5.6%)	DES	2
Total	44			44	18			36

### Conventional transthoracic echocardiography

Comparison of echocardiographic variables between studied groups pre-PCI are depicted in (Tables 4, 5). Patients in 2-stent technique group had more severe MR and more dilated LA compared with provisional group. Post PCI the EF did not show any significant changes immediate post CBL intervention. Meanwhile, the mitral inflow velocities and mitral annular diastolic velocity E" showed improvement with significant reduction in LV filling pressure as estimated from E/E' in both provisional and 2-stent technique groups (*P* < 0.03 and *P* < 0.001) respectively. There were no significant immediate changes in the other echocardiographic variables post PCI.

**Table 4:** Echocardiographic measurements pre-PCI

Patient	Provisional *N* = 44	2 Stent techniques *N* = 18	*P* value
TR			0.2
Severe	1(10%)	0	
Moderate	4(9.1%)	2(11.1%)	
Mild	15(34.1%)	5(27.8%)	
Trivial or no	24(56.8%)	11(61.1%)	
MR			0.02
Severe	1(2.3%)	0	
Moderate	4(9.1%)	2(11.1%)	
Mild	21(47.7%)	11(61.1%)	
Trivial or no	18(40.9%)	6(33.3%)	
LA diameter (mm)	32.2 ± 1.4	39.4 ± .1.0	0.03
LA volume(ml/m^2^)	26.2 ± 9.6	28.9 ± 12.7	0.382
ESD (mm)	31.8 ± 5.6	39.6 ± 3.1	0.056
EDD (mm)	45.9 ± 6.7	53.2 ± 4.5	0.046
EF%	55.9 ± 13.2	51.7 ± 9.6	0.068
Septum	8.9 ± 1.5	8.5 ± 2.1	0.356
LVPW	8.5 ± 1.5	9.4 ± 2	0.135
LVM	221.2 ± 58.2	229.8 ± 49.3	0.33
LVMI	122.3 ± 35.3	131.9 ± 24.8	0.192
TR	0.8 ± 0.9	0.4 ± 0.5	0.156
MR	1.8 ± 0.9	0.9 ± 0.6	0.002
Mitral E	71 ± 26.5	79.2 ± 21.4	0.292
Mitral A	51.6 ± 23.1	59.3 ± 24	0.291
mitral E/A	1.8 ± 1.4	1.3 ± 0.9	0.178
DT	159.7 ± 58.8	160.8 ± 5	0.946
e'	8.0 ± 3.4	8.2 ± 4.9	0.889
E/e'	8.9 ± 5.3	9.6 ± 3.5	0.113

LA: left atrium, TR: tricuspid regurgitation; MR: Mitral regurgitation, LVMI: left ventricular mass index; E/E’ early mitral inflow velocity to early mitral annular velocity; DT: Deceleration time; S’: peak annular systolic velocity; e’ annular early diastolic velocity; a’: peak atrial diastolic velocityك ESD: End-systolic diameter, EDD: End-diastolic diameter,: EF: Ejection fraction, LVPW: left ventricle posterior wall, S/PW: septal to posterior wall ratio; LVM: Left ventricular mass, LVMI: Left ventricular mass index

**Table 5:** Changes in EF and diastolic function following CBL intervention

	2- Stent techniques *N* = 18		*P* value	Provisional *N* = 44		*P* value
	Pre PCI	Post PCI		Pre PCI	Post PCI	
EF%	55.9 ± 13.2	56.4 ± 12.4	0.534	51.7 ± 9.6	52.9 ± 7.5	0.745
Mitral E	71 ± 26.5	65 ± 13.3	0.134	79.2 ± 21.4	66.2 ± 3.4	0.112
Annular e'	8.0 ± 3.4	9.4 ± 4.8	0.02	8.2 ± 4.9	9.1 ± 2.7	0.02
E/e'	8.9 ± 5.3	6.9 ± 2.4	0.01	9.6 ± 3.5	7.2 ± 1.6	0.001

EF: Ejection fraction, Mitral E: early diastolic mitral inflow velocity; e': mitral annular early diastolic velocity, E/e': the ratio of mitral inflow early diastolic velocity to the annular early diastolic velocity using TDI

### Two-dimensional Strain analysis

In provisional group (pre-PCI) impaired PLS was most pronounced for RCA territory followed by LAD and LCX territories. While in 2-stent technique group both impaired LAD and RCA territories were the most pronounced followed by LCX territory.

PCI of the bifurcation lesion positively impacted myocardial function. There was significant improvement in LAD territory, (*P* < 0.001), RCA territory (*P* < 0.01) and GLS (*P* < 0.01). The magnitude of change (**Δ)** post PCI was significantly higher in LAD myocardial segments (34.5%) compared with **Δ** LCX 9.6% and Δ RCA (25.4%), *P* < 0.001 and *P* < 0.01 respectively.

In 2-stent technique group, there was significant improvement of PLS of LAD territory (*P* < 0.01), RCA territory (*P* < 0.01) and GLS (*P* < 0.01) respectively. The improvement in LCX vascular territory did not reach statistical significance in both provisional and 2 stent technique groups.

**Fig. 1:**
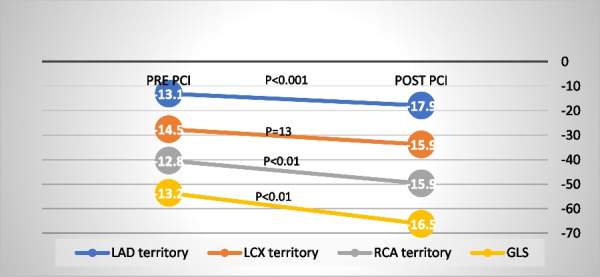
Changes in LV strain post PCI in provisional group

**Fig. 2:**
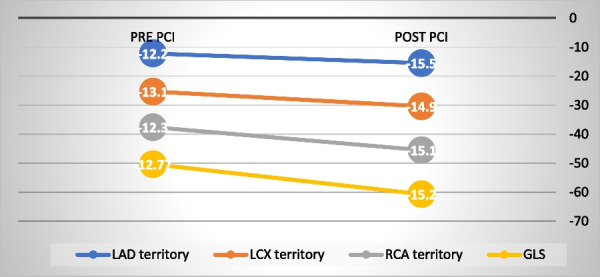
The changes in LV strain post PCI in 2 stent technique

The magnitude of change post PCI was significantly higher in LAD myocardial segments (27.1%) compared to **Δ** LCX (13.7%), *P* < 0.001 and Δ RCA (22.7%), p = 0.213 respectively. The magnitude of change of myocardial function was higher for LAD territory in provisional group compared to the 2- stent technique group (Table 6, Fig. 1,2).

**Table 6:** Left ventricular peak longitudinal systolic strain in studied groups pre and post bifurcation intervention

	Provisional *N* = 44			*P* value	2- Stent techniques *N* = 18			*P* value
	PLS Pre- PCI	PLS Post-PCI	Δ PLS (%)		PLS Pre -PCI	PLS Post -PCI	ΔPLS (%)	
LAD Territory	− 13.1 ± 3.6	− 17.9 ± 4.8	34.5 ± 12	0.001	− 12.2 ± 3.5	− 15.5 ± 5.5	27.1 ± 6	0.01
LCX territory	− 14.5 ± 3.5	− 15.9 ± 3.4	9.6 ± 3	0.334	− 13.1 ± 5.69	− 14.9 ± 6.4	13.7 ± 14	0.154
RCA territory	− 12.8 ± 4.6	− 15.9 ± 5.2	25.4 ± 13	0.01	− 12.3 ± 3.8	− 15.1 ± 3.6	22.7 ± 5	0.01
Apical cap	− 12.2 ± 5.5	− 17.5 ± 5.2	43.4 ± 5	0.001	− 11.5 ± 1.8	− 13.5 ± 3.7	17.3 ± 10	0.01
GLS	− 13.2 ± 4.2	− 16.5 ± 4.5	25.0 ± 7.1	0.01	− 12.77 ± 2.7	− 15.2 ± 4.2	22.1 ± 9	0.01

PLS: peak longitudinal strain; LAD: left anterior descending, LCX: left circumflex; RCA: right coronary artery; PCI: percutaneous coronary intervention; GLS LV global longitudinal strain

**Fig. 3:**
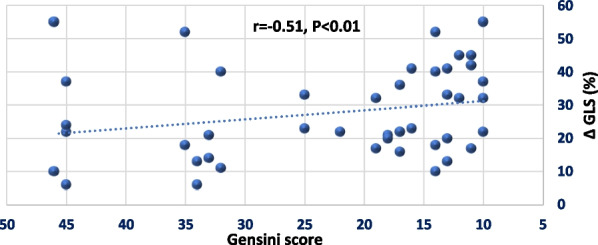
Scatter plot, of GENSINI score and **Δ** GLS in provisional group

**Fig. 4:**
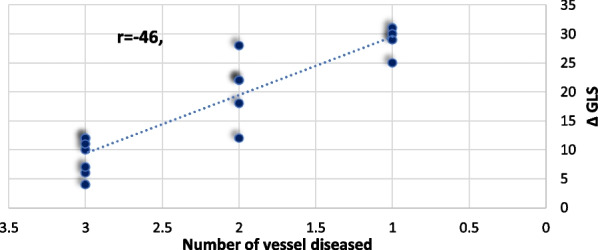
Scatter plot, number of vessel diseased and **Δ** GLS in 2-stent technique group

Using Pearson correlation coefficient, the relationship between the magnitude of change in LV GLS (**Δ**) and all demographic, clinical and echocardiographic variables were analyzed. From all these variables **Δ** GLS was correlated only to the severity of atherosclerosis. In provisional group **Δ** GLS was negatively correlated to Gensini score (r = − 0.51, *P* < 0.01) while in 2 stent technique group **Δ** GLS was negatively correlated to the number of vessel diseased (r = − 0.46, *P* < 0.02) respectively (Figs. 3, 4). No significant relation was observed between change in GLS and any other clinical or echocardiographic variables.

## Discussion

Of all percutaneous coronary procedures, 15–20% are associated with coronary bifurcation lesions (CBLs). The intricacy of these lesions is the cause of poor early and late procedural results. Interventional cardiology's hottest issues include refining bifurcation stenting methods and assessing how they affect patient outcomes in clinical settings. In this study, we used 2D strain imaging to evaluate the impact of several commonly used bifurcation stenting procedures on LV mechanical parameters. The chief results of this study comprise the following: 1-both provisional and 2-stent techniques in CBLs result in favorable improvement of LV deformation as assessed using speckle tracking echocardiography. 2-The planned one-stent strategy (provisional technique) remains preferable to the two-stent strategy for the treatment of bifurcation lesions (71% of CBL treated during elective PCI). However, one stent and two stents strategies allow immediate appraisal of improvement especially in LAD bifurcation lesions.

In our study, despite more severe CAD (as assessed by Gensini score and higher prevalence of anterior MI) in patients subjected to the 2-stent strategy according to the operator preference, successful procedure positively impacted the patient outcome and was capable of improving myocardial function. During the more complex 2-stent technique where stent distortions are more prominent, speckle tracking echocardiography is a preferred tool to accurately evaluate the procedure success.

As far as we are aware, this is the first study to evaluate LV mechanical changes after bifurcation lesion PCI and to compare the impact of one stent and 2 stent strategies on myocardial function using speckle tracking echocardiography.

An innovative echocardiographic method for evaluating global cardiac function is GLS using 2D-STE. This method is based on detecting the distinctive speckle patterns that the myocardium's ultrasound beam interference produces. It does not require specific equipment, and CMR [24] has been used to validate its accuracy. According to Kalam et al., the LVEF indicates that the subendocardial longitudinal fiber is more susceptible to myocardial ischemia and that the reduced LV longitudinal function developed earlier than aberrant radial contraction [25]. The subendocardial longitudinal fiber movement is denoted by GLS [25, 26] therefore, in the case of patients with bifurcation lesion and preserved LV EF%, GLS assessed by 2DSTE is a fairly appropriate method to evaluate the alteration of myocardial function following elective PCI.

One of the interesting findings in our study is the immediate improvement detected in vascular territories post PCI especially the LAD territories (in provisional group: 34% increase in peak systolic strain at territorial level and 25% in GLS and in 2-Stent technique: group 27% in LAD territory and 22% in GLS).

This agrees with previous studies: Ryo et al. [27] that showed improvement of LV function as evaluated by GLS in 35 patients one month after PCI.

Antoni et al. [28] examined LV function following recent myocardial infarction using GLS during one-year follow-up. Patients were described as improvers if there is an increase in GLS ≥ 10%. In the current study, the improvement in GLS following PCI was higher ranged between 22 and 25% in the two groups of patients despite failure of LV EF%, using conventional method, failed to demonstration any change.

The findings of the current study support both the implementation of specific technical steps during coronary stenting and adding myocardial imaging following bifurcation PCI. LV functional assessment documented that appropriately selected techniques for CBL resulted in added favorable outcomes, and 2D strain imaging was able to identify successful revascularization.

In the domain of interventional cardiology, the complicated bifurcation lesion subgroup has drawn a lot of interest and several stenting methods have been devised. The most often used of these is the provisional stenting approach, however in some subgroups of complicated CBLs, the elective double stenting (EDS) procedure is preferable.

In the majority of bifurcation situations, single-stent methods are still considered the gold standard, with provisional or, less frequently, inverted provisional representing the primary operational alternatives [22, 27, 29].

Both procedures may be accomplished by selecting stents sized 1:1 according to the distal vessel to be stented, engaging the main vessel for the standard provisional approach and the side vessel for the inverted provisional. Using multimodal imaging they evidently demonstrate that MV stent being fully expanded, deploying the latest generation drug-eluting stent and optimal apposition after POT. Furthermore, side-cell expansion is encouraged by kissing balloon, to attain optimum results for both main and SB vessels. The metallic coverage of the main vessel was less in the inverted provisional procedure, where a smaller stent size was implanted in the MV, than in the traditional technique. This was the fundamental distinction between the two approaches [22, 27, 29].

Due to its effectiveness and safety in comparative trials, the double kissing crush method may be the recommended EDS approach; nonetheless, it is a multi-step process that requires training. Recently, a number of novel procedures have been introduced to the EDS methods to improve stent scaffolding and decrease unfavorable outcomes, both early and late.

In the particular context of intricate 2-stent techniques, because of the incidence of more mechanical injuries, more prolonged ischemic time and more contrast, we hypothesized that the beneficial effect of myocardial revascularization might be less if compared to one stent provisional technique.

It should be taken into consideration that Beyond the method itself, a multitude of other parameters, including as clinical, demographic, anatomical, and physiological aspects, as well as adjunctive procedural approaches, operator experience, and adjunctive medication, may influence the success of a patient undergoing CBL stenting.

The current study demonstrates and confirms the effectiveness of the provisional approach as the primary strategy when facing complex bifurcation lesions, as one stent approach can improve myocardial function immediately post intervention and even to greater extent in LAD CBL intervention.

TRYTON trial [30] showed that the more frequent minor periprocedural MIs in a bifurcation 2-stent technique, as opposed to the usual 1-stent provisional strategy, prevented the strategy from meeting the noninferiority TVF target. At the 9-month follow-up, it was linked to a decreased stenosis of the SB using the provisional technique. With little incidence of cardiac arrest, stent thrombosis, and clinically indicated revascularization, both approaches proved to be safe. For both arms, there was a difference between angiographic restenosis and clinically driven TVR, suggesting that clinical expression of SB angiographic restenosis is rare. The bifurcation 2-stent method did not provide any benefit in smaller SBs, but it may have some effect in bigger SBs, according to a post-hoc study of SB size and TVF.

In addition to lowering the risk of myocardial infarction and cardiac mortality, percutaneous coronary intervention (PCI) significantly lessens the severity of angina and the need for further operations in patients with maintained left ventricular function who are receiving appropriate medical treatment [31, 32]. The majority of studies thoroughly examine how primary PCI affects cardiac function; however, it is unclear how earlier research on elective bifurcation lesions affects either the diastolic or systolic performance of the left ventricle [33–36].

In prior randomized trials, most planned 2-stent techniques have been found to be inferior to PS in non-LM bifurcation lesions, primarily because of greater periprocedural myonecrosis with multiple stents (and higher rates of stent thrombosis in some studies) [10–13, 37]

By contrast, in the present study of a routine planned 2-stent approach versus provisional in patients with CBL lesions, both techniques resulted in evident improvement of myocardial systolic and diastolic function. This result encourages an appropriate selection of PCI strategy whether one stent or two stent strategy dependents on the anatomical standpoint, operator's preference and skills. The higher rate of early and late adverse events with provisional stenting compared with DK crush stenting in one study published by Chen et al. [14] may in part relate to the anatomy of the distal LM segment. The present study aimed to assess the improvement of left ventricular systolic function after elective CBL PCI using tissue Doppler 2D- strain imaging.

Of note, notwithstanding there was no difference in the rate of clinically relevant periprocedural MI between the 2 techniques, using an analogous definition as that used in the EXCEL trial [8] and recommended by the Society of Cardiac Angiography and Interventions, which has been correlated with subsequent mortality. However, there was a higher rate of periprocedural myonecrosis with both stent techniques, especially the DK crush stenting compared with PS [39]. Lower periprocedural biomarker elevation levels, on the other hand, do not seem to be prognostically significant; nevertheless, additional research is needed in this area when dealing with complicated bifurcation lesions like LM PCI.

These results align with two original meta-analyses that included wholly ongoing randomized trials comparing the 2-stent technique with the provisional approach, which found an increase in periprocedural MIs but no differences in death, revascularization need, or stent thrombosis between the two strategies [40, 41]. These outcomes might be explained by aggressive lesion preparation, greater coronary instrumentation, particularly in smaller SBs, and longer procedure timeframes.

Nevertheless, there were no clinically noteworthy adverse effects, such as cardiac mortality or the requirement for revascularization, as a result of this rise in periprocedural MIs. Similarly, there was no therapeutic advantage from the bifurcation stent strategy's good angiographic outcomes at the 9-month follow-up in SB diameter stenosis. These results highlight the well-known disparity, if a firm edge level of the surrogates is not gotten, between angiography (diameter stenosis severity) and biologic (CK-MB and troponin elevation) surrogate endpoints compared with hard clinical endpoints in coronary PCI clinical trials [42–48].

### Study limitations

There are many limitations in the current study that should be recognized. First, the small sample size and the smaller number of patients with CBLs treated with EDS technique in relation to those treated with PS technique. However, we carefully selected the CBL cohort and excluded all confounders that may alter myocardial mechanical properties. Second, we assessed mainly the myocardial systolic deformation using speckle tracking, a study of diastolic function by strain and strain rate imaging could be considered in further studies. However, diastolic function was assessed using mitral inflow, and TDI and E/e' which well correlated and reflect LV filling pressure especially immediate changes following intervention. Third, only longitudinal LV function was interrogated, and we did not assess circumferential and radial strain, whereas longitudinal function constitutes more than 70% of cardiac performance which better reflects the overall cardiac mechanics. Finally. intermediate and long-term effect of bifurcation stenting techniques were not investigated with patient long term follow up that warrant further investigation.

## Conclusion

Both bifurcation stenting techniques (Provisional and 2 stent techniques) have a considerable positive effect on LV mechanics, early following intervention, as evaluated by 2D‐strain echocardiography. Provisional stenting technique remains the gold standard for treatment of coronary bifurcation lesions. However, the proper choice of the bifurcation stenting technique according to the clinical and anatomical characteristics leads to immediate functional improvement.

## Data Availability

The datasets used and/or analyzed during the current study are available from the corresponding author on reasonable request.

## Abbreviations

CAD: Coronary artery disease
PCI: Percutaneous coronary intervention
CBL: Coronary bifurcation lesion
LV: Left ventricle
EF: Ejection fraction
GLS: Global longitudinal strain
LAD: Left anterior descending
LCX: Left circumflex
RCA: Right coronary artery
CBL: Coronary bifurcation lesion
SB: Side branch
MB: Main branch
MI: Myocardial infarction

## Declarations

### Consents for publication

A written consent for publication was taken from the patients participating in this study including their approval for publication.
